# Clifford Wavelet Entropy for Fetal ECG Extraction

**DOI:** 10.3390/e23070844

**Published:** 2021-06-30

**Authors:** Malika Jallouli, Sabrine Arfaoui, Anouar Ben Mabrouk, Carlo Cattani

**Affiliations:** 1 LATIS Laboratory of Advanced Technology and Intelligent Systems, Université de Sousse, Ecole Nationale d’Ingénieurs de Sousse, Sousse 4023, Tunisia; jallouli.malika3@gmail.com; 2 Laboratory of Algebra, Number Theory and Nonlinear Analysis, Department of Mathematics, Faculty of Sciences, University of Monastir, Avenue of the Environment, Monastir 5019, Tunisia; Sabrine.arfaoui@issatm.rnu.tn (S.A.); Anouar.benmabrouk@fsm.rnu.tn (A.B.M.); 3 Department of Mathematics, Faculty of Sciences, University of Tabuk, Tabuk 47512, Saudi Arabia; 4 Department of Mathematics, Higher Institute of Applied Mathematics and Computer Science, University of Kairouan, Street of Assad Ibn Alfourat, Kairouan 3100, Tunisia; 5 Engineering School (DEIM), Tuscia University, Largo dell’Università, 01100 Viterbo, Italy

**Keywords:** ECG, abdominal ECG, fetal ECG, wavelets/multiwavelets, Clifford wavelets/multiwavelets, Haar–Faber–Schauder wavelets/multiwavelets, entropy, 42C40, 92C55

## Abstract

Analysis of the fetal heart rate during pregnancy is essential for monitoring the proper development of the fetus. Current fetal heart monitoring techniques lack the accuracy in fetal heart rate monitoring and features acquisition, resulting in diagnostic medical issues. The challenge lies in the extraction of the fetal ECG from the mother ECG during pregnancy. This approach has the advantage of being a reliable and non-invasive technique. In the present paper, a wavelet/multiwavelet method is proposed to perfectly extract the fetal ECG parameters from the abdominal mother ECG. In a first step, due to the wavelet/mutiwavelet processing, a denoising procedure is applied to separate the noised parts from the denoised ones. The denoised signal is assumed to be a mixture of both the MECG and the FECG. One of the well-known measures of accuracy in information processing is the concept of entropy. In the present work, a wavelet/multiwavelet Shannon-type entropy is constructed and applied to evaluate the order/disorder of the extracted FECG signal. The experimental results apply to a recent class of Clifford wavelets constructed in Arfaoui, et al. *J. Math. Imaging Vis.* 2020, 62, 73–97, and Arfaoui, et al. *Acta Appl. Math.* 2020, 170, 1–35. Additionally, classical Haar–Faber–Schauder wavelets are applied for the purpose of comparison. Two main well-known databases have been applied, the DAISY database and the CinC Challenge 2013 database. The achieved accuracy over the test databases resulted in *Se* = 100%, *PPV* = 100% for FECG extraction and peak detection.

## 1. Introduction and Motivation

According to annual WHO statistics, cardiovascular disease is considered as one of the major causes of the death in the world (See https://www.who.int/news-room/fact-sheets/detail/the-top-10-causes-of-death/ (accessed on 28 December 2020)). Therefore, the diagnosis of these diseases is always a vital task. In hospitals’ cardiology departments, the electrocardiogram signal remains one of the predominant and most widely used tools for the diagnosis and analysis of cardiac arrhythmia. See also [1].

In reality, ECG examination may be a non-invasive tool performed by bio-physicians to explore the functioning of the heart by the use of external electrodes brought into contact with the skin. It is a signal that reflects the electrical activity of the heart. It informs about how the heart works by measuring its electrical activity. In fact, with each heartbeat, an electrical impulse (or wave) passes through the heart. This wave causes a contraction of the heart muscle so that it expels blood from the heart. The ECG measures and records the electrical activity that passes through the heart permitting one to decide whether the electrical activity observed is normal or abnormal. Although ECG examination may be painless, and non-invasive, its interpretation remains complex, and requires methodical analysis, and some clinical experience. It highlights various cardiac anomalies, and has an important place in diagnostic examinations in cardiology, as for coronary artery disease. See for example [2,3,4].

On the other hand, the FECG signal reflects the electrophysiological activity of the fetal heart. Congenital heart defects originate in early stages of pregnancy, when the heart is still in the formation stage, and they can affect any of the parts or functions of the heart. Cardiac anomalies may occur due to a genetic syndrome, inherited disorder, or environmental factors, such as infections or drug misuse [5,6,7,8]. Fetal abnormalities may be detected during fetal development in time by analyzing the fetal ECG waveform.

The FECG is a crucial clinical issue for monitoring the development and well-being of the fetus, throughout pregnancy and childbirth. The challenge is to be able to reliably extract, from external and non-invasive sensors positioned on the mother’s abdomen, an FECG signal of sufficient quality to allow clinical diagnosis. The main difficulty lies in the fact that the abdominal ECG (AbdECG) signal of a pregnant woman is a mixture of several signals (MECG, FECG and noise due to uterine contractions, artefacts by movements of the fetus and the mother), and that the FECG is of lower energy compared to other present signals.

In this paper, a wavelet/multiwavelet method is proposed to extract the FECG parameters from the MECG. The proposed approach is based on the extraction of significant parameters from the MECG signal reconstructed by suitable wavelets/multiwavelets. From the reconstructed signal, the existing forms of noise are eliminated and the parameters related to the FECG are detected.

The ability to estimate the frequency spectrum of signals as a function of time makes it useful in some cases of ECG processing. Indeed, in medicine, the ECG of a sick patient is obviously different from that of a healthy one. However, this difference is sometimes very difficult to spot when the EKG is given as a function of time. It becomes evident when it is given as a function of the frequency. The inconvenient is that the mathematical tool applied, which is based on Fourier series gives the quantity of each frequency present in the signal for the whole observation period. Fourier transform, therefore, becomes ineffective for a signal whose frequency spectrum varies considerably over time. Unlike the Fourier analysis, wavelet analysis offers a wide range of basic functions from which a flexible choice of the most appropriate analyzing mode for a given application is possible.

Now, we briefly recall the second concept to be used consisting of Shannon’s entropy. It is well-known that entropy measure is one of best tools to obtain an optimal reconstruction of signals from a basis of information. The concept of entropy has been introduced in fact very earlier in the 18th century, where researchers applied it for evaluating the order/disorder of the information contained in a system based on probability theory. Next, the concept of entropy has been widely spread and applied in many fields, such as statistical physics in studying microscopic behavior of systems, in computer science, information processing, topological entropy, Kolmogorov–Sinai metric entropy [9], and dynamical systems in mathematics. In the literature, many variants have been already acted, such as Rényi and Kolmogorov [10,11,12]. Guido et al. proposed in [13] a shapelets based entropy for the analysis of discrete-time signals. In [14], the authors served of Daubechies wavelets to construct an entropy measure for biomedical images efficiency proof. See also [15,16,17,18].

These studies motivate us to adapt entropy measure to evaluate the order/disorder of the extracted FECG signal based on wavelets. Our aim is to construct a variant of Shannon’s entropy by using wavelet/multiwavelet coefficients [19,20,21,22]. This will permit us to evaluate, in a precise and automatic way, the optimal reconstruction level of signals, that best represents the reality.

In wavelet theory, indeed, the concept of entropy is very known nowadays, and has also been applied widely. In this context, wavelet theory permits the extraction of information on the frequency content while preserving the localization in order to obtain a time/frequency or space/scale representation of the signal. The entropy measure will be used as an accuracy and efficiency tool of wavelet algorithms and thus to measure the stability of the methods/algorithms developed.

In summary, this work has several purposes. From a theoretical mathematical point of view, the present work falls within the framework of the application of wavelets in bio-signal processing. Essentially, it aims to prove the efficiency of Clifford wavelets against classical ones and thus, to show that Clifford wavelets may be good candidates for signal processing. Recall that the problem of choosing the best wavelets in practical tasks is always and already persistent and ambiguous. Secondly, the present work also aims to introduce a new form of Shannon’s entropy to measure the order/disorder of the extracted and/or reconstructed signals from the noisy ones relative to the wavelet/multiwavelet application. From a practical point of view, the present work considers the problem of extracting the FECG signal from the MECG one and the localization of eventual peaks especially for the FECG. Such a problem is still a challenge for biologists. Two databases of ECG signals are used in order to show the effectiveness and/or the performance of the method. Measurements through Se and PPV are applied for such a goal on DAISY and CinC Challenge 2013 databases.

This paper is organized as follows. Section 3 is a brief state of the art of the most common FECG extraction methods. CinC Challenge 2013 database methods are essentially reviewed. Section 4 is concerned with wavelet and multiwavelet presentation. The basic steps in the construction of the wavelets/multiwavelets to be applied in the present work are recalled briefly, such as Haar and Faber–Schauder wavelets and their associated multiwavelet, and the Clifford wavelets and their associated multiwavelets. A brief comparison between these classes of wavelets is provided. Section 5 is concerned with the presentation of our methodology applied in the present study. Firstly, the wavelet/multiwavelet processing of signals is briefly described. Next, the Shannon-like entropy based on wavelets/multiwavelets is introduced. The section is achieved by the description of the FECG extraction using wavelets/multiwavelets, and the entropy measure of the optimal order, followed by the relative measure of the accuracy and sensitivity. Section 6 is devoted to the development of the bio-experimentation due to the wavelet/multiwavelet processing of ECG signals in order to extract the FECG from the MECG. The experiments proved the effectiveness of the proposed multiwavelet method in extracting the FECG signal. Additionally, the superiority of Clifford wavelets/multiwavelets as recent variants in wavelet theory is shown compared to the classical HFSch class of wavelets/multiwavelets. Section 7 presents the conclusions, in which a brief review of the results developed in our present work is provided with some eventual future directions.

## 2. FECG Extraction Brief Review

The FECG, which is believed to contain more information than conventional ultrasound methods, is always measured by electrodes on the mother’s abdomen. However, the recorded signal always suffers from the mixture of several sources of noise and interference including the very high level of the MECG. In previous studies, several methods have been proposed for extracting the ECG from signals recorded by electrodes placed on the surface of the mother’s body. Despite technological improvements, extracting FECG from abdominal recordings is still a difficult problem that has been addressed by a large number of studies. However, due to the low signal-to-noise ratio of these signals, the application of FECG was limited to the analysis of heartbeats and invasive ECG recordings during childbirth.

In [23], the authors proposed extracting the fetal electrocardiogram from a single-lead maternal abdominal ECG. The algorithm is composed of three components. First, the maternal and fetal heart rates are estimated by the deshaped short time Fourier transform, which is a recently proposed non-linear time-frequency analysis technique. The beat tracking technique is the second component, which is applied to accurately obtain the maternal and fetal R peaks. The third component consists of establishing the maternal and fetal ECG waveforms by the non-local median.

The authors of [24] presented an extended non-linear Bayesian filtering procedure for extracting ECG from a single channel, as encountered in the fetal ECG extraction from abdominal sensor. The recorded signals are modeled as the summation of several ECG signals. Each of them is described by a non-linear dynamic model.

The present study also joins the one developed in [25], where the authors applied wavelets for assessing fetal cardiac rhythms from abdominal ECGs.

In [26], a cancelation method for the maternal ECG has been developed based on a combination of maternal QRS detection, heart rate, interval selection, fiducial points location inside this interval, superimposition of the intervals, calculation of the mean signal, and its sequential subtraction from the whole FECG.

Di Maria et al. in [27] developed a robust algorithm for analyzing abdominal FECG, and testing its performance in the Computing in Cardiology Physionet Challenge 2013 by combining frequency filtering and wavelet denoising.

In [28], the authors served of the CinC Challenge database for developing an FECG extraction method from contaminated signals, using a multistage interference, and noise cancellation relative to the time parameter, space, and frequency characteristics of the FECG, and its interference. The proposed method joins both temporal and statistical properties of ECG signals.

In [29], the CinC Challenge 2013 database has been applied to recognize the fetal heart rate and its RR intervals from non-invasive fetal electrocardiogram signals. Kuzilek and Lhotska proposed in [30] a wavelet technique to FECG analysis based on CinC Challenge database. A wavelet Shannon entropy has been applied for peak detection.

In [31], a multilead component regression approach has been investigated for maternal ECG removal and multichannel correlation-based FHR detector. Liu and Li investigated in [32] a wavelet method for multiple purposes due to an FECG signal from CinC Challenge database, such as FHR estimation, RR, and QT intervals.

Maier and Dickhaus proposed a dynamical hybrid method in [33] combining information from an arbitrary number of abdominal channels into a virtual channel and estimating the local RR interval and initial fetal QRS positions. Next, a dynamic programming is applied to refine the obtained positions relative to SNR, signal amplitude, and RR interval continuity.

Niknazar et al. [34] investigated the detection of fetal QRS due to multichannel ECG signals containing both fetal and maternal ECGs, using tensor decomposition. Akhbari et al. [35] combined the tensor decomposition method with extended Kalman filters for the extraction of the FECG signal. See also [36,37] for a similar study.

In [38], the authors proposed a non-invasive fetal QRS detection algorithm based on fetal ECG source signal enhancement. The maternal QRS are preceded by a reduction in noise allowing their detection to be possible and next followed by fetal QRS localization. See also [39,40,41,42] for linear transformations methods and statistical ones.

Plesinger et al. in [43] developed an automatic extraction method of the R-wave positions of children from non-invasive multichannel FECG records due to the CinC Challenge 2013 database. The method consists of eliminating the mother’s heartbeats from the total signal, defining the presumed multichannel shape of the R-wave for the child, and its record-in recognition. See also [44,45].

In [46], a multistep method based on the so-called semi-blind source separation technique is applied for the separation of the ECG sources and R-peaks. Additionally, the FECG is estimated by the same technique. Starc in [47] used the Cinc Challenge database to apply filtering methods combined with shifting, and scaling for non-invasive fetal multilead RR interval determination (See also [48,49,50,51]).

In the present research, the objective is to improve the signal processing methods used in fetal cardiographs, and to provide efficient solutions to this problem, by developing suitable techniques for extracting and filtering ECG signals from the fetuses recorded by an array of electrodes placed on the mother’s womb. So, for a better extraction of ECG waveforms from the fetus in order to aid in the medical diagnosis of cardiac pathology, the approach envisaged consists of improving the estimation of the FECG signal using two wavelet/multiwavelet-based methods such as the one developed in [52] and consisting of the simplest wavelet/multiwavelet toolkit, and the last recent one developed in [53,54] due to Clifford wavelets as the most recent forms in the field.

## 3. Two Wavelet/Multiwavelet Processors

In this section, principal tools to be applied in our study, consisting of wavelets and their extension to multiwavelets, are recalled.

Wavelet analysis appeared in the early 1980s as a multidisciplinary tool that brought together engineers, mathematicians and physicists. The mathematical synthesis led to new results, which brought broader perspectives in each original discipline. By this time, most scientific researchers had heard of wavelets.

Wavelets originated when certain subjects of study required frequency and time analysis simultaneously. In the nineteenth century, Fourier analysis was the only technique allowing the decomposition of a signal into frequencies’ components. Unfortunately, it provides a frequency analysis but does not allow the temporal localization, especially for abrupt changes. See for instance [22,55,56,57,58,59,60].

Fourier analysis is based on the fact that functions showing periodicity and certain degree of regularity can be represented by a linear combination of sines and cosines. The coefficients of this linear combination provide information at the level of the frequencies present in the signal.

Multiwavelets have been introduced since the early 1990s as another view of wavelets permitting us to re-write wavelet analysis in a vector form ([61,62]). The majority of cases of existing multiwavelets’ constructions, especially in experimental cases, starts from one wavelet or scaling function ψ/φ and consider the vector

Ψ=(ψ(.),ψ(.−1),⋯,ψ(.−N))orΦ=(φ(.),φ(.−1),⋯,φ(.−N)),

where *N* is the corresponding filter length associated with such functions. This view of wavelets has even though some advantages, such as short supports, smoothness, accuracy, symmetry, and orthogonality. However, it surely induces some correlation between the components of multiwavelet decomposition of signals due to the non-independence of the multiwavelet components, especially in non-orthogonal case. In the present paper, some types of multiwavelets will be applied differently, where the components are issued each one from a different source. One of them has been already applied in [63] and has shown to be powerful in estimating biomedical signals. A second variant is due to Clifford wavelets, recently constructed in [53,54]. Such wavelets will be shown to be able to induce in a natural way a variant of multiwavelets by considering their Clifford components such as the real parts, the vector parts, the bi-vector parts, etc., as wavelets, and merge them to obtain a multiwavelet.

In a first step, an improvement of wavelet processing is recalled by applying recent families of multiwavelets issued from single ones, where independent components for multiscaling and multiwavelet mother functions are used. Vector-valued mother multiwavelets due to [52,53,54,63] are considered, such as $\Psi_{HFSch}=\left(\psi_{H},\psi_{FSch}\right)$ for the case of Haar–Faber–Schauder multiwavelet essentially issued from [52], and $\Psi_{Cl}=\left(\psi_{1},\psi_{2}\right)$ for the case of Clifford multiwavelets due to [53,54]. These will be recalled with brief details in the next subsections.

### 3.1. The Haar–Faber–Schauder System

Recall that the Haar mother wavelet ($\psi_{H}=\chi_{\left[0,1/2\right]}-\chi_{\left[1/2,1\right]}$) is the most simple case in explicit wavelets. It resembles piece-wise constant signals and it has been shown to cover many situations in signal processing. It is compactly supported, not enough regular, explicit, oscillating with one vanishing moment. It yields an orthonormal system ${\left(\psi_{H}^{j,k}\right)}_{j,k\in Z}$, where $\psi_{H}^{j,k}\left(t\right)=2^{-j/2}\psi_{H}\left(2^{j}t-k\right)$. More importantly, it is simple to implement. It is adapted more to piece-wise constant (may be periodic) signals ([64]).

However, this system may not be well-adapted to approximate more complex cases such as piece-wise linear ones for example. In this case, better systems may be adapted. The second system known in functional approximation is the piece-wise linear Faber–Schauder wavelet system based on the mother wavelet

$$2\psi_{FSch}\left(x\right)=\Lambda \left(2x\right)-2\Lambda \left(2x-1\right)+\Lambda \left(2x-2\right),$$

where Λ(x)=max(0,1−|x|). Such a system has been also proved to be suitable in many situations in signal processing (see for example [65]). The Faber–Schauder wavelet also presents many advantages, and important features, such as the simple explicit mathematical form, compact support, and orthogonality. These advantages have been encouraging the work of [52], where the authors have developed an entropy-based procedure for approximating signals with such wavelets by considering a multiwavelet case; its components are exactly Haar and Faber–Schauder wavelets. In the present work, an exploitation of such a case is also considered by choosing the Haar–Faber–Schauder multiwavelet $\Psi_{HFSch}={\left(\psi_{H}\;\psi_{FSch}\right)}^{T}$, where the upper script ${}^{T}$ stands for the transpose. This multiwavelet merges the characteristics of both Haar and Faber–Schauder systems and thus constitutes a better loop for the processing of signals. It is also compactly supported, explicit, and has a reduced number of non-zero coefficients, obtained by recursively averaging and differentiating.

### 3.2. Clifford Wavelets and Multiwavelets

In this subsection, a brief review of Clifford-valued wavelets, and multiwavelets constructed on the real Clifford algebras is provided. For the convenience, the focus will be on the simple case $R_{3}$ and the useful tools for the associated wavelet analysis. Details may be found in [53,54]. Consider the Euclidean space $R^{3}$ with its canonical basis B=(i,j,k), and equipped with an interior product defined on the basis by

$$i^{2}=j^{2}=k^{2}=-1\;\mathrm{and}\;ij+ji=ik+ki=jk+kj=0.$$

Next,

$$e_{1}=ij,\;e_{2}=ik,\;e_{3}=jk,\;\mathrm{and}\;e_{4}=ijk.$$

The real Clifford algebra $R_{3}$ is the R-algebra with dimension 8, the basis of which is $\tilde{B}=\left(1,i,j,k,e_{1},e_{2},e_{3},e_{4}\right)$. Any element $u\in R_{3}$ is written as

$$u=\underset{\mathrm{real}\;\mathrm{part}}{\underset{︸}{u_{0}}}+\underset{\mathrm{vector}\;\mathrm{part}}{\underset{︸}{u_{1}i+u_{2}j+u_{3}k}}+\underset{\mathrm{bivector}\;\mathrm{part}}{\underset{︸}{v_{1}e_{1}+v_{2}e_{2}+v_{3}e_{3}}}+\underset{\mathrm{trivector}\;\mathrm{part}}{\underset{︸}{v_{4}e_{4}}}.$$

In the sequel, a conjugation rule will be applied such as

$$\bar{u}=u_{0}-u_{1}i-u_{2}j-u_{3}k-v_{1}e_{1}-v_{2}e_{2}-v_{3}e_{3}+v_{4}e_{4}.$$

On the Clifford algebra $R_{3}$, a function $f:R^{3}⟶R_{3}$ will be expressed as

$$f\left(x\right)=f_{0}\left(x\right)+f_{1}\left(x\right)i+f_{2}\left(x\right)j+f_{3}\left(x\right)k+{\tilde{f}}_{1}\left(x\right)e_{1}+{\tilde{f}}_{2}\left(x\right)e_{2}+{\tilde{f}}_{3}\left(x\right)e_{3}+{\tilde{f}}_{4}\left(x\right)e_{4},$$

where the $f_{l}$ and the ${\tilde{f}}_{l}$, l=0,1,2,3 are real-valued functions on $R^{3}$.

One of the concepts used to construct wavelets on the real Clifford algebra $R_{3}$ is the notion of monogenicity, based on the Dirac operator

$$\partial_{x}=\partial_{x_{1}}i+\partial_{x_{2}}j+\partial_{x_{3}}k.$$

and the Cauchy–Kowalevski extension (CK-extension). A function $f=f(x_{1},x_{2},x_{3})$ is said to be monogenic on $R^{3}$ if $\partial_{x}f=0$. The CK-extension permits us to extend *f* to a Clifford-valued function on $R^{4}$ by

(1) $$F\left(x_{0},x\right)=\exp\left(-x_{0}\partial_{x}\right)f\left(x\right)=\sum_{k=0}^{\infty }\frac{{\left(-x_{0}\right)}^{k}}{k!}\partial_{x}^{k}f\left(x\right).$$

Exploiting the fact that *F* is monogenic, Clifford-valued wavelets are constructed. One reason for this is due to the fact that Clifford wavelets are the last variants of wavelet functions developed by researchers in order to overcome many problems that are not well-investigated by classical transforms. The challenge iof such concepts is not the wavelet functions themselves, but also the structure of Clifford algebras, and their flexibility to include different forms of vector analysis in the same time. There are, in the literature, two main methods to construct Clifford wavelets. The first one is based on the spin group, which includes the factor of rotation in the wavelet analysis, provided with the translation and dilation factors—see [17,66,67,68,69,70]. The second is based on monogenic polynomials. These ones constitute natural extensions of orthogonal polynomials to the case of Clifford algebras. Recall that orthogonal polynomials are widely applied in wavelet theory and signal processing. See, for example, [66,71,72,73,74,75,76,77,78,79,80,81,82,83,84,85,86,87].

In the present work, the construction conducted in [53,54] will be applied, where a class of Clifford–Hermite–Jacobi wavelet functions have been introduced by considering the Clifford weight

$$\omega_{\alpha ,\beta }\left(\underline{x}\right)=\left(1+\right|\underline{x}{{|}^{2})}^{\alpha }e^{-\beta |\underline{x}{|}^{2}},\;\underline{x}\in R_{3}.$$

This leads to a Clifford mother wavelet

$$\psi_{\ell }^{\alpha ,\beta }\left(\underline{x}\right)=P_{\ell ,m}^{\alpha +\ell ,\beta +\ell }\left(\underline{x}\right)\omega_{\alpha ,\beta }\left(\underline{x}\right),$$

where the $P_{\ell }^{\alpha ,\beta }\left(\underline{x}\right)$ are the Clifford polynomials generated from the CK extension (1) of $\omega_{\alpha ,\beta }$, which may be expressed as

$$F^{*}\left(t,\underline{x}\right)=\sum_{\ell =0}^{\infty }\frac{t^{\ell }}{\ell !}P_{\ell }^{\alpha ,\beta }\left(\underline{x}\right)\;\omega_{\alpha -\ell ,\beta -\ell }\left(\underline{x}\right).$$

By fixing α=1.5, β=α−1 and taking the vector parts, the following mother Clifford wavelets are obtained,

$$\psi_{1}\left(\underline{x}\right)=C_{1}\left(-2\right|\underline{x}\left|+\right|\underline{x}{|}^{3})(1+|\underline{x}{{|}^{2})}^{3/2}e^{-|\underline{x}{|}^{2}/2}i,$$

$$\psi_{2}\left(\underline{x}\right)=C_{2}\left(\right|\underline{x}\left|+16\right|\underline{x}{|}^{3}+24|\underline{x}{|}^{5}+13|\underline{x}{|}^{7}+|\underline{x}{|}^{9})(1+|\underline{x}{{|}^{2})}^{3/2}e^{-|\underline{x}{|}^{2}/2}i,$$

where the $C_{j}^{\prime }$s (j=1,2) are normalization constants with respect to the $L^{2}$-norm, and i=(1,0,0). See [53,54] for more details on the original construction of these wavelets. These will be considered as two-order multiwavelets by considering $\Psi_{Cl}={\left(\psi_{1}\;\psi_{2}\right)}^{T}$.

### 3.3. Brief Comparison between the Two Classes of Wavelets/Multiwavelets

Each class of the two wavelets/multiwavelets recalled above has its advantages. The first one is compactly supported, piece-wise linear and permits a reduced number (2 or 3) of non-zero coefficients, sufficient to cover the experiment. The Clifford wavelets/multiwavelets are highly regular, with Gaussian decay, which permits some artificial compactness of the support and thus joins the first one in some characteristics. Additionally, Gaussian decay also helps overcome the boundary problems usually confronted in such studies. Moreover, the computation of the total number of the filters coefficients to conduct a multiwavelet analysis is not necessary.

Associated filters such as Gabor and Clifford–Gabor, Hermite and Clifford–Hermite are already developed and proved to be localized in both the spatial and frequency domains. Such localization is a basic fact in signal processing as it is responsible for the measurement of local structures such as points, lines, and edges, in order to facilitate subsequent interpretation of these structures at higher stages (known as high-level vision). More details and facts are developed in [88,89] with applications related to signal processing, compression, and quality. See also [90].

## 4. Methodology

In the present section, the purpose is to describe our methodology. It is globally based on three steps. The first one is due to the wavelet/multiwavelet processing, in which wavelets/multiwavelets are applied to separate the approximation parts (usually called the denoised parts) of the signals from the noisy ones. The second step consists of evaluating the entropy measure to conclude on the accuracy of the wavelet/multiwavelet method as well as its sensitivity.

### 4.1. Wavelet/Multiwavelet Processing

Let $X_{t}$, t≥0 be a signal with finite energy. Applying wavelets and/or multiwavelets in the signal processing results for a level *J* of decomposition in a number of positions *k*. Denote

(2) $$AX_{t}^{J}=\sum_{k}C_{J,k}\phi_{J,k}\left(t\right)$$

its wavelet/multiwavelet approximation at the level *J* relatively to wavelet/multiwavelet multiresolution analysis, where the $C_{J,k}$ are the wavelet/multiwavelet scaling or approximation coefficients of $X_{t}$ evaluated as

(3) $$C_{J,k}=\int X_{t}2^{J/2}\phi \left(2^{J}t-k\right)dt,$$

where ϕ is the (multi)scaling function associated to the multiresolution analysis. Similarly, the wavelet/multiwavelet detail component of $X_{t}$ at a level *j* is

(4) $$DX_{t}^{j}=\sum_{k}d_{j,k}\psi_{j,k},$$

where $d_{j,k}$ is the wavelet/multiwavelet detail coefficient at the level *j* and the position *k*, evaluated as

(5) $$d_{j,k}=\int X_{t}2^{j/2}\psi \left(2^{j}t-k\right)dt,$$

where ψ is the mother wavelet/multiwavelet of the multiresolution. The decomposition of the signal $X_{t}$ at the level *J* is written as

(6) $$X_{t}^{J}=AX_{t}^{J}+\sum_{j=0}^{J}DX_{t}^{j}.$$

When applying a two-order multiwavelet such as the Clifford multiwavelet $\psi_{Cl}$ introduced above, a first component $A_{J}^{1}$ is obtained for each level *J*, corresponding to an approximation at the level *J* according to the first component of the two-order multiwavelet, a second component $A_{J}^{2}$ corresponding to an approximation at the level *J* according to the second component of the two-order multiwavelet and next a superposition of detail components $D_{j}^{1}$ and $D_{j}^{2}$ (0≤j≤J) corresponding to the first and the second components of the analyzing multiwavelet, respectively. As a result, for the case of two-order multiwavelet decomposition at a level *J*, it holds that

(7) $$S_{J}=A_{J}^{1}+A_{J}^{2}+\sum_{j=0}^{J}D_{j}^{1}+\sum_{j=0}^{J}D_{j}^{2}.$$

The task consists of applying two (but blind each one against the other) cameras, each inducing an independent representation which can be noisy for the other, and next superposing these two representations to attenuate the noise resulting from each one, and then have a new and final performant image. The operation resembles the method of installing two surveillance cameras, for example, to cover the maximum space, and thus induce a complete image.

To resume, for a two-dimensional signal $S={\left(S_{1},S_{2}\right)}^{T}$, such as the one to be tackled in the present paper, the detail component at a level *J* of decomposition is

(8) $$DS_{J}=\sum_{l}D_{J,l}\Psi_{J,l},$$

where the multiwavelet coefficients $D_{J,l}$ are (2,2)-matrices. The sum of these detail components induces the approximation of the signal at the level *J* as

(9) $$AS_{J}=\sum_{j<J}DS_{j}.$$

As a consequence, the signal *S* may be approximated at the level *J* as

(10) $$S_{J}=AS_{J}+DS_{J}.$$

Using (9), the last approximation may be written as

(11) $$S≃DS_{J}+DS_{J-1}+DS_{J-2}+\cdots +DS_{0}+AS_{0}.$$

The principal problem in wavelet/multiwavelet information processing is the fixation of a prior optimal reconstruction level that represents the closest model to the original data. This problem is always subjective and in most cases approximated recursively by iterated algorithms. The information extracted is considered as optimal when a desired relative error (fixed a priory) is reached. For example, in wavelet processing, such an error is evaluated by the $L^{2}$-norm of the difference signal between the experimental or observed signal and the approximated one.

### 4.2. Wavelet/Multiwavelet Entropy

In this section, the purpose is to show that the entropy measure may be a good processor to obtain an optimal approximation of the data. The assessment of the wavelet entropy will allow us to determine in a precise way the optimal order of reconstruction. Recall indeed that the entropy in its general form in both mathematical and physical points of view is a type of dimension. Therefore, it should be, somehow, a global measure of invariance for the studied system. Its value will tend to a stability as the multiresolution level increases.

Shannon’s entropy is introduced to measure the randomness or the order/disorder of the information contained in a system. It also permits us to calculate the minimum amount of data required to describe such a system without loss of information [91,92]. In [93], entropy-based algorithms have been derived to select from a wavelet packet library the best basis to express the information well. In [94,95], the concept of entropy has been applied in the same direction as previously conducted to extract, from a discrete wavelet packet, substantial information about turbulent flow fields. In [96], some experimental scenarios have been applied based on wavelet entropy for an optimal scale search and coherent secondary flow characterization. In [97], Rosso developed a wavelet entropy method called spectral entropy to measure the complexity of a system by measuring the homogeneity of the spectral distribution of a signal from its discrete wavelet transform. Mathematically, given a probability $p_{i}$ of a particle *i* to occur in some situation in the system, the Shannon entropy of the whole system is evaluated as

(12) $$\mathrm{Entropy}=-\sum_{i}p_{i}\log p_{i}.$$

In the present section, a modified variant of Shannon’s entropy will be applied. For *j* fixed as a level of approximation, a notion of wavelet/multiwavelet energy at the level *j* is defined as

(13) $$EX_{t,j}=\sum_{k}{∥C_{j,k}∥}^{2},$$

where the $C_{j,k}$ are the approximation coefficients relative to the wavelet/multiwavelet multiresolution at the position *k* and the level *j*. The total energy of the signal is evaluated as

(14) $$EX_{t}=\sum_{j}EX_{t,j}.$$

Next, a probability distribution of energy is introduced, which describes in some sense the contribution of the *j*-level approximation $X_{t,j}$ of $X_{t}$ in its total energy. Let

(15) $$p_{j}=\frac{EX_{t,j}}{EX_{t}}.$$

It may be also understood as the probability or the frequency of the presence of a particle in the box (j,k) relative to the basis element $\left(\varphi_{j,k},\psi_{j,k}\right)$. Next, the wavelet/multiwavelet entropy analogue to Shannon’s is simply obtained by replacing, in (12), the probability $p_{i}$ by the one evaluated in (15). In practice, of course, a finite *J*-estimation of such entropy has to be computed as

(16) $$Ent\left(J\right)=-\sum_{j=0}^{J}p_{j}\log p_{j},\;J\in N.$$

Our aim in the present work is to show that this entropy is a good measure that informs us about the ‘best’ (‘optimal’) approximation of the FECG.

Indeed, a basic problem raised in optimal reconstruction aims is how to define automatically and accurately the optimal reconstruction level. In this section, multiwavelet entropy will be shown to be a good method permitting one to stop the procedure with the best approximation guaranteed. The optimal level of reconstruction of a signal using multiwavelets is evaluated by the evaluation of the error between the original signal and the reconstructed one. The optimal level is the one that offers the lowest error. On the other hand, the multiwavelet entropy approach allows the detection of the optimal order for the reconstruction during the decomposition phase, without going to the reconstruction itself, which proves its efficiency in front of classical error estimates.

Assessment of the multiwavelet entropy allows one to determine in a precise way the optimal level of reconstruction. Indeed, as entropy in its general form and definition as well as mathematical/physical meaning is a type of dimension. Therefore, it should be, somehow, a global measure of invariance for the studied system. Its value should therefore be stationary or quietly constant as the multiresolution level increases.

### 4.3. The FECG Extraction

In the present section, multiwavelets are applied for the extraction of FECG signal. The explicit HFSCH multiwavelet introduced in [52,63] will be served as classical processor, and the Clifford one developed recently in [53,54], and recalled previously as explicit Clifford wavelet.

Now, a description of the wavelet/multiwavelet processing of the ECG, FECG and MECG signals will be exposed. The abdominal ECG signal is a compound signal containing both the mother own ECG, the fetal ECG, and the noise,

(17) AbdECG=MECG+FECG+Noise.

At a decomposition level *J*, the noise has to be eliminated so that,

(18) $$AbdECG_{J}=AMECG_{J}+DMECG_{J}+AFECG_{J}+DFECG_{J}.$$

In ECG processing, it is well-known that the MECG signal is widely stronger with regard to time than the FECG signal embedded in it. Moreover, the noises in which the FECG is embedded are also stronger. Therefore, it is natural that the energy of the MECG signal is the highest while the energy of the FECG signal is the lowest. This will allow the multiwavelet approximation coefficients of the decomposed signal to be easily separated, and thus the FECG can beextracted.

**Figure 1:**
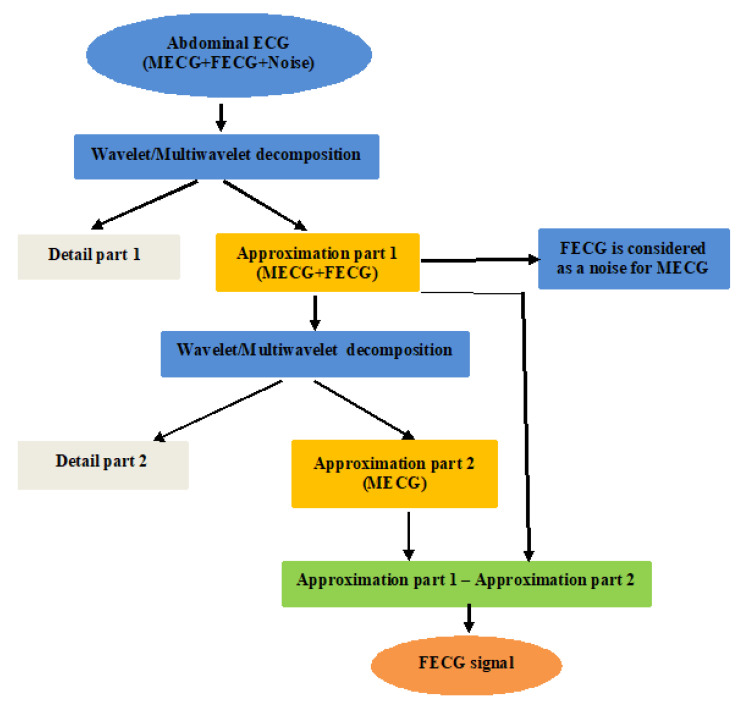
The multiwavelet FECG extraction principle.

The diagram in Figure 1 illustrates the principle of FECG extraction using the multiwavelet method. The extraction of FECG from the wavelet MECG is based on the principle of thresholding and/or the separation of mixed signal components. The phenomenon looks like a mixed signal composed of many sinusoids with different frequencies and amplitudes, and mixed with some noises. A first filtering eliminates the noise, and extracts the periodic or pseudo-periodic part of the total signal, which is theoretically composed of the sum of the sinusoids, and thus propagates with the lower frequency. The second phase consists of separating the sinusoids based on the fact that their wavelet coefficients, and thus their frequency spectra, form separated bands.

The approximation and detail projections of the FECG signal will be thus extracted as

(19) $$\left\{\begin{array}{c} AFECG_{J}=AAbdECG_{J}-AMECG_{J}\\ \mathrm{and}\\ DFECG_{J}=DAbdECG_{J}-DMECG_{J}. \end{array}\right.$$

Finally, the concept of thresholding and peak detection is used to detect the R-peaks of the FECG signal. An overview of our method is summarized in Algorithm 1. The true positive peaks, as well as false positive, and false negative are localized, and thus used in the computation of the measurements of accuracy and performance.
**Algorithm 1 FECG extraction****Input** AbdECG
**Output** FECG
**Begin**
[Detail1,App1] = Wavelet/multiwavelet decomposition(AbdECG);
MECG = Wavelet/multiwavelet reconstruction(App1);
[Detail2,App2] = Wavelet/multiwavelet decomposition(MECG);
App3 = App1-App2;
FECG = Wavelet/multiwavelet reconstruction(App3);
**Return** FECG
**End**


The pre-processing is an initial filtering step. Indeed, the real AbdECG signal is a mixture of various signals and noises due to the breathing movements of the patient, the change in position of the instrument, the interaction between the electrodes and the skin, etc. These factors may cause a drift from the baseline, which is considered as a low frequency noise. It affects the usefulness of the ECG signal, and consequently, the clinical evaluation. Depending on the noise present, a necessary filtering should be performed. Such a filter accepts only frequencies between 5 and 20 and stops the rest. The so-called pre-processed signal is obtained. The resulting signal will be used as AbdECG for the next steps. Readers may refer to [98] for the subject of pre-processing ECG signals.

### 4.4. Performance Measurements

In order to assess the accuracy of our method, especially the detection of the positive real peaks, we used, as in the existing methods, the most used measures such as the fetal heart rate (FHR), the positive predictive value (PPV), and the sensitivity (Se). There are, in fact, other measures that may be applied, such as the Challenge’s scoring system based on the mean squared error between smoothed and re-sampled versions of the reference FHR, and the root mean square difference of corresponding RR intervals. The FHR is evaluated as

(20) $$FHR=\frac{\mathrm{Number}\mathrm{of}\mathrm{peaks}\mathrm{detected}}{\mathrm{Duration}\mathrm{of}\mathrm{signal}}*60.$$

The *FHR* gives a clear idea of the arrhythmias and other abnormalities attained by the fetus. The accuracy, the Positive predictive value (PPV), sensitivity (Se) and, $F_{1}$-measure introduced in [37] are estimated, respectively, by

(21) $$Accuracy=\frac{TD}{TD+FP+FN}*100,$$

(22) $$PPV=\frac{TD}{TD+FP}*100,$$

(23) $$Se=\frac{TD}{TD+FN}*100,$$

and

(24) $$F_{1}=2*\frac{PPV*Se}{PPV+Se}.$$

where *TD* is the truly diagnosed (or true positive peak), *FP* is the false positive peak and *FN* is the false negative peak.

## 5. Results

In the experimental part, the main purpose is to apply our method to basically two different bases. In the first, an abdominal electrocardiogram signal is applied issued from the DAISY database [99]. Next, in order to show the performance of the method, and to compare with other cases, we applied it on signals extracted from the well-known CinC Challenge 2013 database.

### 5.1. Data Collection and Bases

The most important task in such type of studies is the quality, and the availability of data to be used. Recording data with high quality may be affected by several factors, such as the number of electrodes applied, their type, heterogeneity of the patient population, conditions, and gestational ages, noise causes, and factors, sampling frequency, amplitude resolution, etc.

For FECG data, for example, the possibility of being invasive or non-invasive is always present for collection methods of data. In fact, it is noticeable that invasive collected signals have better quality than those collected via non-invasive methods. However, the main drawback may be explained by the fact that invasive methods may only apply an intra-uterine electrode during labor, where the recording electrodes are in direct contact with the fetal skin. This is one of the main inconveniences for invasive methods for collecting FECG data.

Non-invasive methods apply, however, signals recorded from the maternal abdomen, in any stage of pregnancy. They use a high number of electrodes. Non-invasive recording of FECG signals also present a main drawback due to their low signal-to-noise ratio.

The main databases used in the present work are essentially the Database for the Identification of Systems, abbreviated as DAISY dataset, and the Computing in Cardiology Challenge 2013, abbreviated as Cinc Challenge 2013. The DAISY database [99] (http://homes.esat.kuleuven.be/~smc/daisy/12-28-2020) consists of a single dataset of cutaneous potential recording of a pregnant woman.

The second database consists of non-invasive FECG multichannel abdominal recordings, as part of the PhysioBank [100] (https://archive.physionet.org/challenge/2013/04-19-2021). The 2013 PhysioNet/CinC Challenge attracted a total of 53 teams trying to non-invasively extract fetal ECG information from maternal abdominal leads. Most teams used a two-step approach, where the first step aimed to remove the maternal QRS, followed by a second step to extract the fetal QRS. Removal of the maternal component was achieved using techniques including subspatial decomposition or reconstruction ([28,30,31,32,39,45,101]), adaptive filtering and averaging ([33,34,36,38,40,41]), wavelet denoising ([43,46,47,98,102]) and a fusion of several approaches [37]. The final step in the detection of fetal QRS uses various approaches, including adapted filtering ([31,33,43]), detection of Christov beats [26], entropy ([30,31]), RS slope [103], expectation weighting [104], echo state recurrent neural network [40], or merging of several [42] methods. Behar et al. [37], instead of relying on a single technique for maternal ECG extraction, decided to implement a merger of several different extraction methods. Another successful approach has been the use of adaptive QRS models for the mother, fetus, or both, thus allowing realistic non-stationary conditions [36].

### 5.2. The Processing of DAISY Signals

In this part, an abdominal electrocardiogram signal is applied that is issued from the DAISY database [99]. It contains three channels recorded signals for 10 s time intervals. The proposed method is implemented using MATLAB software.

**Figure 2:**
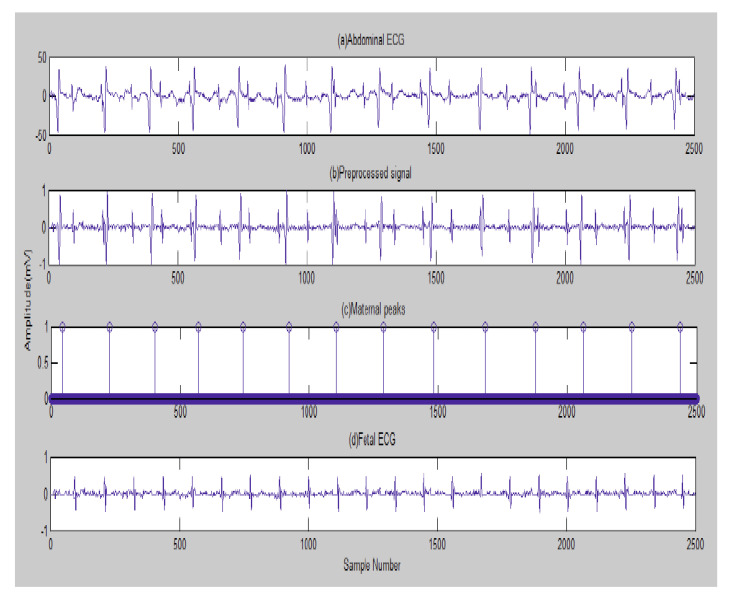
Identification of maternal peaks and MECG removal [105]—DAISY.

The first result is illustrated graphically by Figure 2 due to the implementation of the existing method in [105]: (a) the channel 2 AbdECG; (b) pre-processed signal; (c) maternal peaks; and (d) FECG.

**Figure 3:**
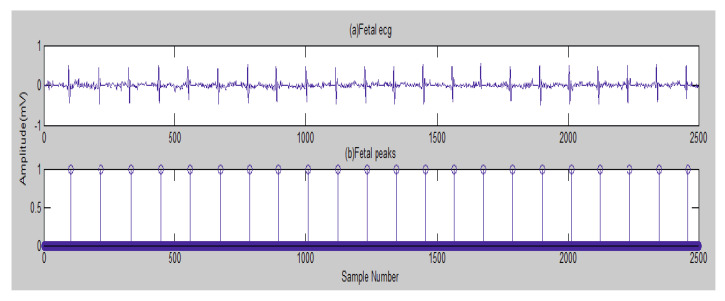
The FECG and its detected peaks [105]—DAISY.

Next, in Figure 3, the FECG peaks detected are indicated. A fetal heart rate of 132 bpm (beats per minute) was obtained for channel 2. The normal range of FHR lies between 120 and 160 bpm.

The real peaks, which are detected, are truly diagnosed (TD) peaks. Some peaks which are detected, although they are actually not true, are categorized as false positives (FPs). An actual peak that is not detected is considered as a false negative (FN) [105].

**Figure 4:**
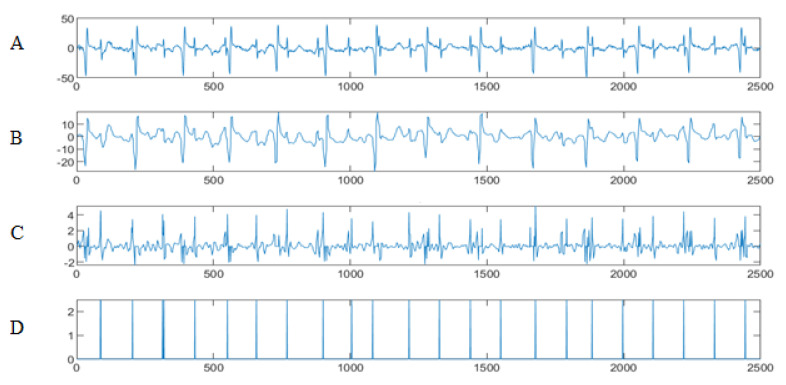
FECG extraction and peak detection using HFSch multiwavelet: (**A**) AbdECG (**B**) MECG (**C**) FECG (**D**) FECG peaks—DAISY.

To test our wavelet/multiwavelet method, and to evaluate its effectiveness, an implementation for channel 2 was conducted. Figure 4 shows the result of HFSCH wavelet/multiwavelet processing for (A) channel 1 AbdECG, (B) the MECG signal, (C) the FECG signal and (D) the FECG peaks.

**Figure 5:**
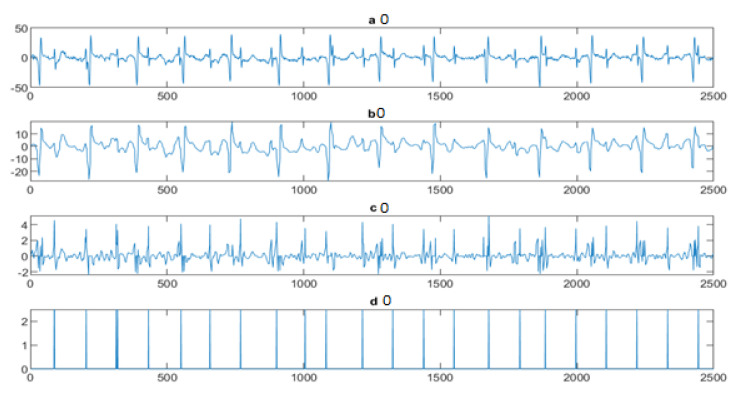
FECG extraction and peak detection using $\psi_{1}$ Clifford wavelet: (**a0**) AbdECG (**b0**) MECG (**c0**) FECG (**d0**) FECG peaks—DAISY.

**Figure 6:**
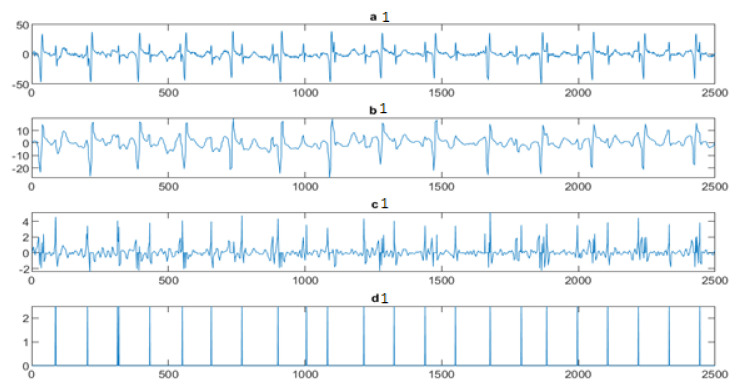
FECG extraction and peak detection using $\psi_{2}$ Clifford wavelet: (**a1**) AbdECG, (**b1**) MECG, (**c1**) FECG, and (**d1**) FECG peaks—DAISY.

**Figure 7:**
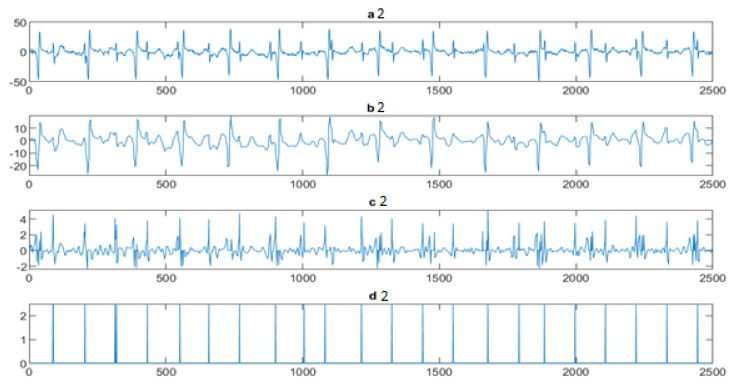
FECG extraction and peak detection using $\Psi_{CL}$ Clifford multiwavelet: (**a2**) AbdECG, (**b2**) MECG, (**c2**) FECG, and (**d2**) FECG peaks—DAISY.

Next, the wavelet/multiwavelet processors continued to act. Figure 5, Figure 6 and Figure 7 illustrate the result of the application of the Clifford wavelets $\psi_{1}$, $\psi_{2}$, and their associated Clifford multiwavelet $\psi_{CL}$.

Next, further assessment of the wavelet/multiwavelet proposed approach is developed on channels 3 and 4 of AbdECG. Table 1 summarizes the results of the R-peaks detected by different methods. Table 2 shows the accuracy results and Table 3 shows the sensitivity results.

**Figure 8:**
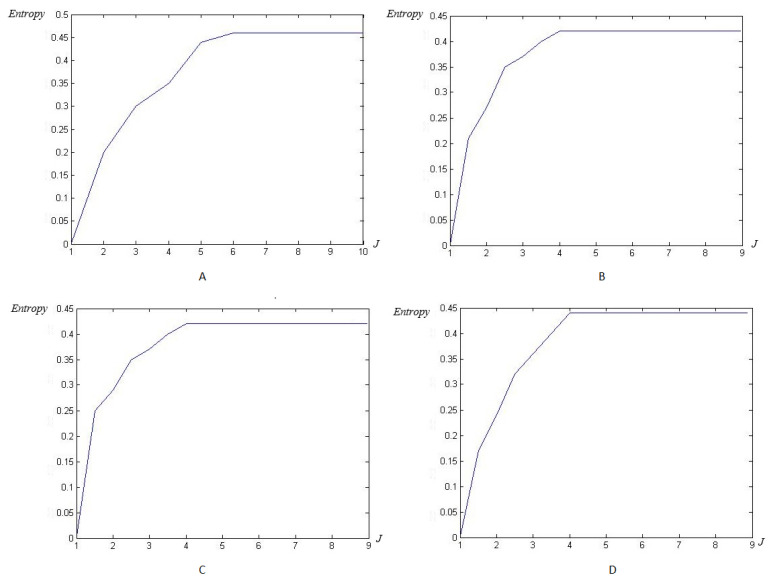
Entropy estimation for FECG signal-DAISY with wavelet/multiwavelet: (**A**) HFSCH multiwavelet entropy, (**B**) $\psi_{1}$-Clifford wavelet entropy, (**C**) $\psi_{2}$-Clifford wavelet entropy, (**D**) $\psi_{CL}$-Clifford multiwavelet entropy.

The next step is to apply our multiwavelet entropy for the ECG signals to prove that such entropy may be an automatic black-box allowing us to reach the optimal reconstruction level rapidly and accurately. In Figure 8: (A) illustrates the classical HFSCH multiwavelet entropy, (B) shows the Clifford wavelet $\psi_{1}$ entropy, (C) illustrates the entropy measure for single Clifford wavelet $\psi_{2}$, and (D) shows the result of entropy estimation using Clifford multiwavelet $\psi_{CL}$.

### 5.3. The Processing of Cinc Challenge Database

In this subsection, the aim is to act our method on the well-known Cinc Challenge 2013 database ([100]), in order to confirm the performance and the efficiency of the method developed for more signals from other bases. The Cinc Challenge 2013 database consists of one-minute fetal ECG recordings, including each one four non-invasive abdominal signals.

Recall that the present method aims to localize fetal QRS by assuring more improvement than existing ones. The accuracy was evaluated using 75 non-invasive four-channel abdominal ECG recordings from the 2013 PhysioNet/Computing in Cardiology challenge. These recordings were made on ten women (healthy and pathological patients), aged between 21 and 33 years (27.1±4.3 years) and between gestational weeks 20 and 28 weeks (25.0±2.5 weeks). No ectopic beat was found for the mother or the fetus. Data were collected by a bipolar probe setup at a sampling rate of 1000 Hz. Each recording had its maternal QRS (MQRS) and FQRS annotated and corrected. In our study, an interval of acceptance was fixed to ±20 ms between the detection and the nearest reference annotation to account for the higher expected heart rate (in the standard, ±150 ms is suggested).

**Figure 9:**
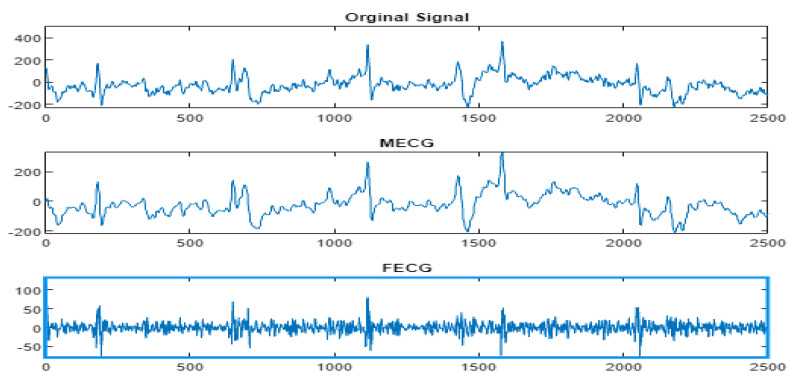
Signal 1 (a12–CinC) FECG extraction using $\psi_{CL}$-multiwavelet.

**Figure 10:**
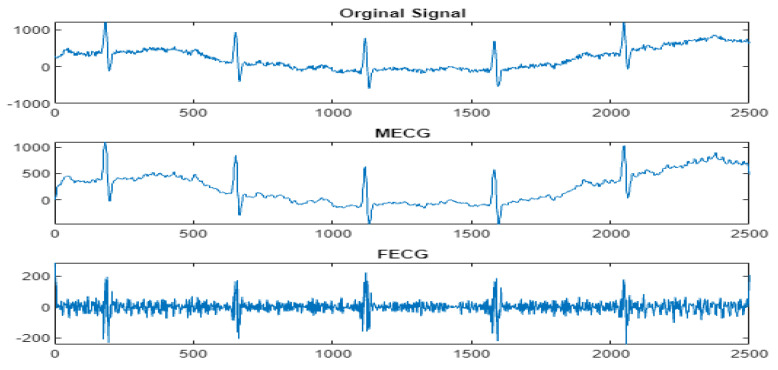
Signal 2 (a29–CinC) FECG extraction using $\psi_{CL}$-multiwavelet.

**Figure 11:**
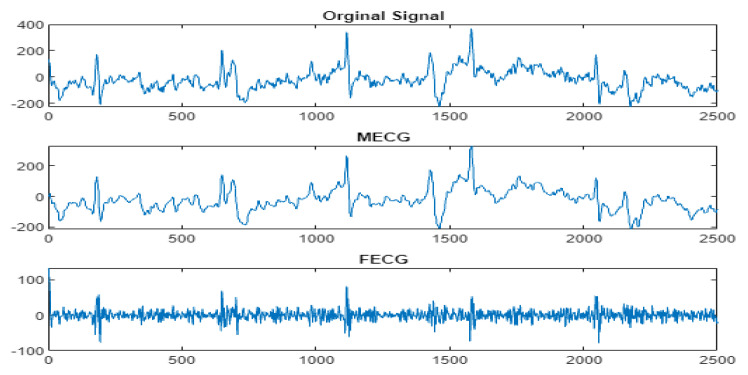
Signal 3 (a47–CinC) FECG extraction using $\psi_{CL}$-multiwavelet.

**Figure 12:**
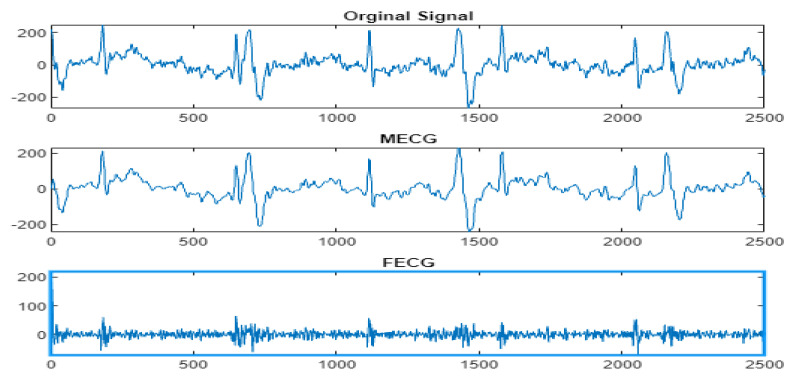
Signal 4 (a59–CinC) FECG extraction using $\psi_{CL}$-multiwavelet.

As in the previous database, the last Clifford multiwavelet yielded the best results, we applied it for the present CinC challenge 2013 database. In our experimental tests, we applied samples from the set-a series; a12, a29, a47, a59, as shown in the corresponding figures and tables. Recall that for a normal ECG, the space between two QRS is always the same regardless of the time of recording. Each P wave must be followed by a QRS. For the detection of peaks, a threshold has been set for each signal according to the maximum frequency for a R-peak. In order to not detect the FP peaks, the additional peaks have been eliminated in the interval RR, so we computed the RR interval, and thus, the false peaks were not taken, they were considered as noise. Figure 9, Figure 10, Figure 11 and Figure 12 illustrate the FECG extraction for the first signal.

**Figure 13:**
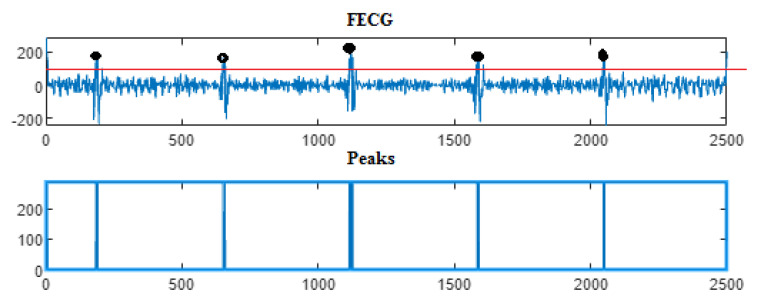
$\psi_{CL}$–Clifford multiwavelet FECG extraction and peak detection for signal a12–CinC.

**Figure 14:**
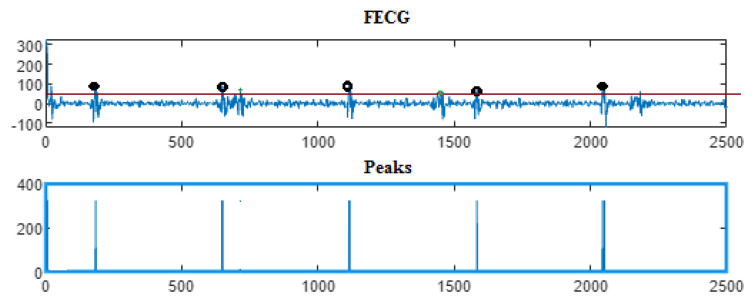
$\psi_{CL}$–Clifford multiwavelet FECG extraction and peak detection for signal a29–CinC.

**Figure 15:**
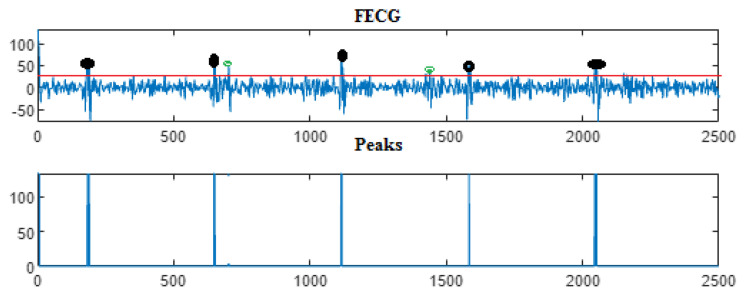
$\psi_{CL}$–Clifford multiwavelet FECG extraction and peak detection for signal a47–CinC.

**Figure 16:**
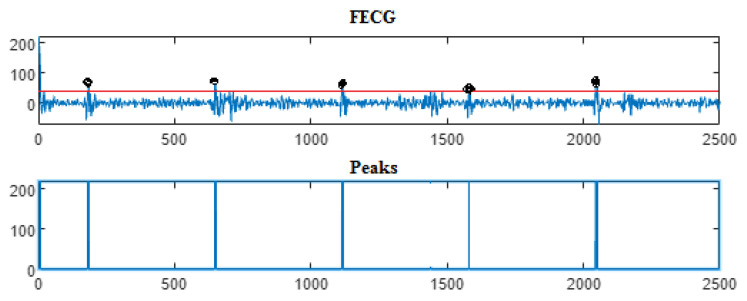
$\psi_{CL}$–Clifford multiwavelet FECG extraction and peak detection for a59–CinC.

Next, as previously mentioned, the relative peaks localization due to the $\psi_{CL}$-Clifford multiwavelet is illustrated in Figure 13 for the first CinC Challenge signal, Figure 14 for the second signal, Figure 15 for the third, and Figure 16 for the fourth CinC Challenge signal. Additionally, for each one of these signals, the accuracy and the sensitivity are provided in Table 4, Table 5 and Table 6, respectively.

**Figure 17:**
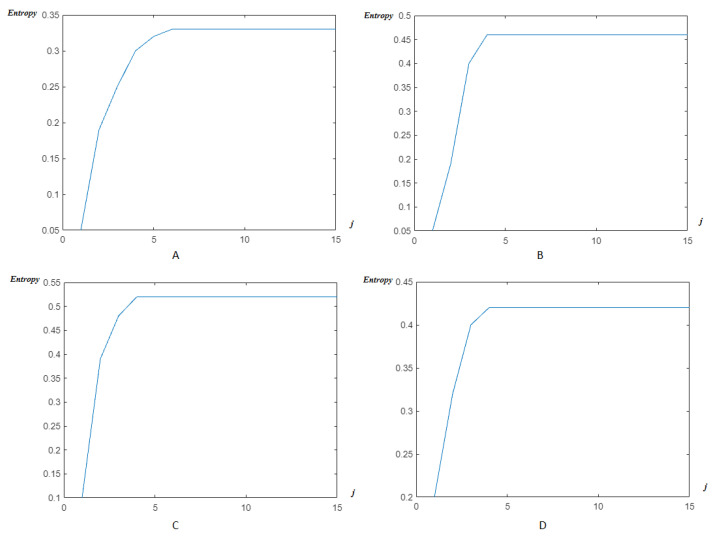
Entropy estimation for FECG a12–CinC with wavelets/multiwavelets: (**A**) HFSCH, (**B**) $\psi_{1}$, (**C**) $\psi_{2}$, and (**D**) $\psi_{CL}$.

**Figure 18:**
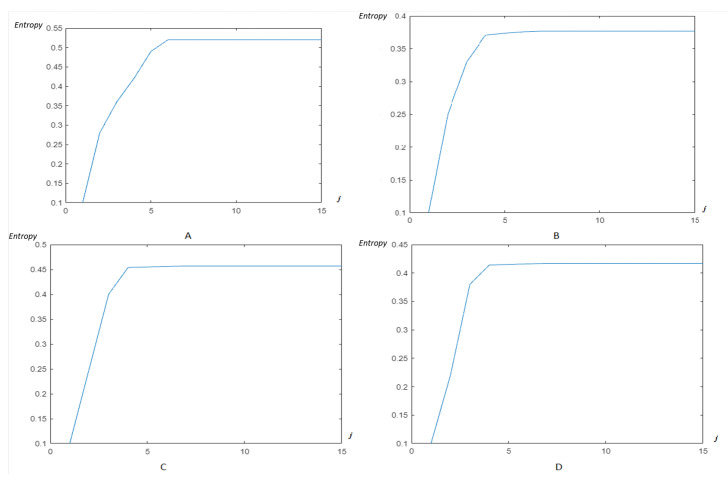
Entropy estimation for FECG a29–CinC with wavelets/multiwavelets: (**A**) HFSCH, (**B**) $\psi_{1}$, (**C**) $\psi_{2}$, and (**D**) $\psi_{CL}$.

**Figure 19:**
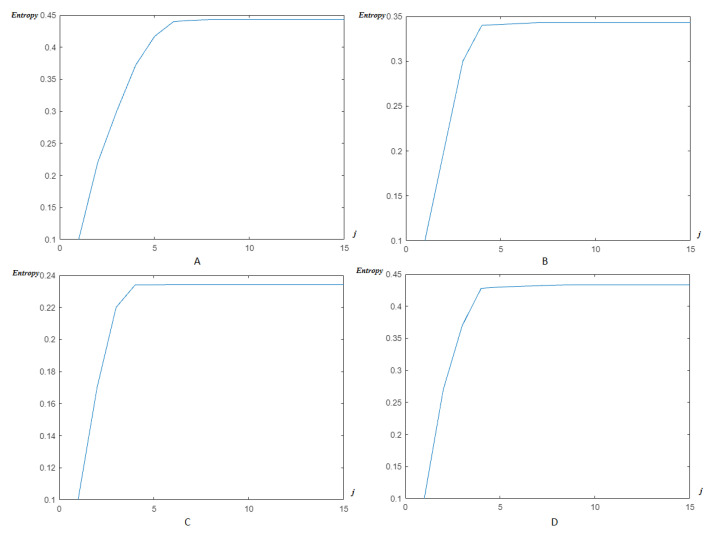
Entropy estimation for FECG a47–CinC wavelets/multiwavelets: (**A**) HFSCH, (**B**) $\psi_{1}$, (**C**) $\psi_{2}$, and (**D**) $\psi_{CL}$.

**Figure 20:**
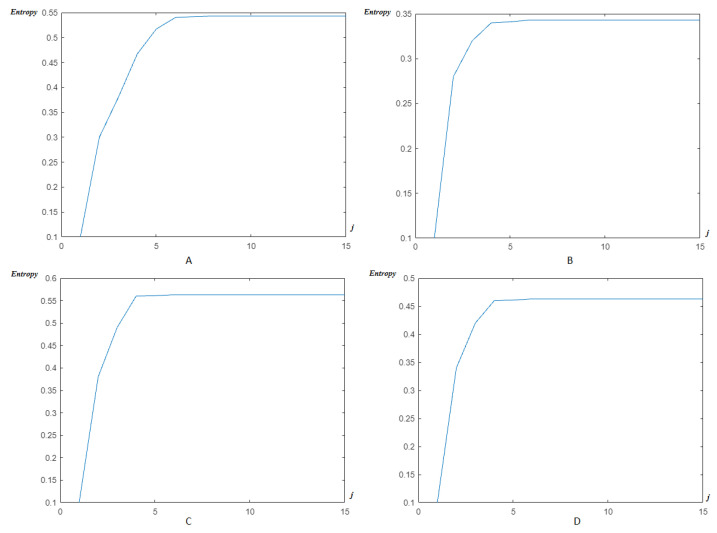
Entropy estimation for FECG a59–CinC wavelets/multiwavelets: (**A**) HFSCH, (**B**) $\psi_{1}$, (**C**) $\psi_{2}$, and (**D**) $\psi_{CL}$.

Now, as previously, the modified Shannon’s entropy developed in the present paper is applied to CinC Challenge 2013 database signals, in order to compare with the previous base, and to show more the performance of the method. In Figure 17, the entropy due to HFSCH multiwavelet (Figure 17A), $\psi_{1}$-Clifford wavelet (Figure 17B), $\psi_{2}$-Clifford wavelet (Figure 17C), $\psi_{CL}$-Clifford multiwavelet (Figure 17D), respectively, are illustrated for the first signal. Figure 18, illustrates the entropy due to the same wavelets/multiwavelets for the second signal. Figure 19, illustrates the entropy due to the same wavelets/multiwavelets for the third signal. Finally, Figure 20 illustrates the entropy due to the same wavelets/multiwa-velets for the last signal in the CinC challenge database.

To achieve the aim of our study, a comparison of both the accuracy and the sensitivity for the two databases used is provided in Table 7 below.

A comparison our study for fetal ECG parameters extraction is also addressed with some of the best studies conducted in CinC Challenge 2013 team, such as [101,106]. The results are summarized in Table 8.

## 6. Discussion

For the DAISY database, in Figure 3, a fetal heart rate of 132 bpm (beats per minute) was obtained for channel 2. The normal range of FHR lies between 120 and 160 bpm.

Next, the eventual peaks were localized, and classified as TD, FP, and FN, for the extraction of the real ones. To test our method, and to evaluate its effectiveness, an implementation for channels 2 was conducted.

Next, in order to further validate our method, and for further assessment, the proposed approach was implemented for channels 3 and 4 of AbdECG. A comparison of the results obtained and those shown in [105] was conducted. Table 1 shows the R-peaks detected by our proposed methods and the one in [105]. It is clear that our approach allow the detection of all the peaks present in FECG signal.

The findings confirm that our proposed method achieved much better results and all R-peaks of the FECG are detected successfully.

Next, by applying the new modified entropy to reach the optimal reconstruction level rapidly, and accurately, the efficiency of such a tool to reach an optimal level of accuracy can be seen in Figure 8. For the classical wavelets, the order is J=8. It decreases next to J=6 for the classical multiwavelets and reached a reduced level J=4 for the Clifford multiwavelets, showing their superiority as recent variants of wavelets.

In fact, a large number of studies applied the DAISY database, although it is criticized for several reasons, such as, the waveform of the different channel signals, where it is noticed that there is almost no delay between two corresponding R-peaks of different channel signals, which may be not reasonable. To confirm and/or to guarantee the performance of the proposed method, a second database was applied, the CinC challenge 2013.

The results due to the second CinC Challenge 2013 database are illustrated firstly in Figure 9, Figure 10, Figure 11 and Figure 12 for the FECG signal extraction from the MECG one. The figures show the ability of our method to achieve such an extraction.

Next, as for the previous case, the localization of relative peaks for each signal issued from the Cinc Challenge database is provided in Figure 13, Figure 14, Figure 15 and Figure 16, for signals 1, 2, 3, and 4, respectively, showing the success of localizing such peaks. Additionally, the accuracy and sensitivity are summarized in Table 3, Table 4 and Table 5, also confirming the success in localizing the peaks of the FECG extracted.

Figure 17, Figure 18, Figure 19 and Figure 20 show that an optimal, and reduced order for the wavelet/multiwavelet method may be reached for a level J=4 for the CinC Challenge 2013 database signals.

Finally, Table 7 and Table 8 confirm, by means of a comparison with existing results, especially the best ones in CinC Challenge 2013, the performance of the present study for both databases in terms of sensitivity, accuracy, intervals of localized and detected annotations, with a 100% percentage for both the Se and the PPV.

## 7. Conclusions

In the present paper, wavelet/multiwavelet processors have been applied for ECG signal processing. Extraction of the FECG signal from the MECG one has been proved to be possible, and efficient, by using two main sets of wavelets/multiwavelets, such as, the Haar–Faber–Schauder system, as the most recent, and simple explicit set, and Clifford wavelets, as newest set of wavelets/multiwavelets constructed by means of Clifford algebras. Moreover, a modified Shannon entropy has been introduced relative to these multiwavelets and also examined on the same experimental examples to show the efficiency of the introduced method. The experiments proved the effectiveness of the Clifford wavelets in front of the classical HFSCH example, although this system has also proved its efficiency in many cases of information processing.

In fact, many aims have been achieved in the present work. Clifford wavelets/multiwa- velets have been shown to be efficient in processing ECG signals, which may be a positive answer to the best choice of wavelet/multiwavelet functions to be used for practical aims. Furthermore, a modified variant of Shannon’s entropy has been constructed to measure the order/disorder of the extracted and/or reconstructed signals. From a practical point of view, the problem of extracting the FECG signal from the MECG one has been investigated, provided with a good localization of eventual peaks of the FECG signal. Such a problem is still a challenge for biologists. Applied to two well-known databases, the proposed study achieved the scores Se=100%, and PPV=100% for the extraction of FECG signal and the localization of its peaks. We intend for our results to be extended and/or applied to other signals for denoising, extracting and/or separating mixed signals. 

## Figures and Tables

**Table 1:** R-peaks detected—DAISY.

Ch. No.	Total pks	Pks det in [105]	HFSch Multiwavelet pks det for FECG	$\psi_{1}$ pks det	$\psi_{2}$ pks det	$\psi_{\mathrm{CL}}$ pks det
2	22	22	22	22	22	22
3	21	21	21	21	21	21
4	21	22	21	21	21	21

**Table 2:** Accuracy (%) with different method—DAISY.

Channel Number	Acc [105]	HFSch Multiwavelet Acc for FECG	Acc Using $\psi_{1}$	Acc Using $\psi_{2}$	Acc Using $\psi_{\mathrm{CL}}$
2	100	100	100	100	100
3	100	100	100	100	100
4	86.95	100	100	100	100

**Table 3:** Sensitivity (%) with different method—DAISY.

Channel Number	Sens [105]	HFSch Multiwavelet Sens for FECG	Se Using $\psi_{1}$	Se Using $\psi_{2}$	Se Using $\psi_{\mathrm{CL}}$
2	100	100	100	100	100
3	100	100	100	100	100
4	95.23	100	100	100	100

**Table 4:** R-peaks detected/CinC.

Sig. No.	Total pks	HFSch Multiwavelet pks det	$\psi_{1}$ pks det	$\psi_{2}$ pks det	$\psi_{\mathrm{CL}}$ pks det
a12	5	5	5	5	5
a29	5	5	5	5	5
a47	5	5	5	5	5
a59	5	5	5	5	5

**Table 5:** Accuracy (%) with different method/CinC.

Sig. No.	HFSch Multiwavelet Acc for FECG	Acc Using $\psi_{1}$	Acc Using $\psi_{2}$	Acc Using $\psi_{\mathrm{CL}}$
a12	100	100	100	100
a29	100	100	100	100
a47	100	100	100	100
a59	100	100	100	100

**Table 6:** Sensitivity (%) with different method/CinC.

Sig. No.	HFSch Multiwavelet Sens for FECG	Se Using $\psi_{1}$	Se Using $\psi_{2}$	Se Using $\psi_{\mathrm{CL}}$
a12	100	100	100	100
a29	100	100	100	100
a47	100	100	100	100
a59	100	100	100	100

**Table 7:** Performance measurment: accuracy, sensitivity (%), PPV (%) and $F_{1}$-measure/DAISY/CinC.

Database	Accuracy (%)	Se (%)	PPV (%)	$F_{1}$
DAISY	100	100	100	100
CinC	100	100	100	100

**Table 8:** Evaluation results of the proposed method for locating both fetal QRS from all 75 aECG recordings compared to best CinC Challenge results [101,106].

Method	TA/[106]	EKS/[106]	SQA + FTM [101]	New Method
TP	-	-	9573	10,088
FP	-	-	639	24
FN	-	-	596	81
Accuracy(%)	96.0±13.4	91.2±23.2	88.57	98.96
Se (%)	97.4±11	93.1±20.3	94.13	99.76
PPV (%)	97.2±10.7	92.8±20.3	93.74	99.2
$F_{1}$	97.1±10.8	93.0±20.3	93.9	99.47

## Data Availability

Not applicable.

## Abbreviations

ECG	Electrocardiogram
MECG	Mother electrocardiogram
FECG	Fetal electrocardiogram
WHO	World Health Organization
AbdECG	Abdominal electrocardiogram
EKG	Electrocardiography
$p_{j}$	The probability at the level *j*
Se	Sensitivity
PPV	Positive predictive value
DAISY	Data base for the Identification of Systems
CinC Challenge 2013	Computing in Cardiology Challenge 2013
R-peaks	Real peaks
STFT	Short time Fourier transform
QRS	ventricular depolarization curve
RR	Interval between two R-peaks
FHR	Fetal heart rate
QT	The sum of depolarization (QRS) and repolarization (T)
SNR	Signal-to-noise ratio
R-Wave	Ventricular depolarization
HFSCH	Haar-Faber-Schauder
TD	Truly diagnosed
FP	False positive
FN	False negative
$AFECG_{J}$	Approximation of the fetal electrocardiogram at the level *J*
$AMECG_{J}$	Approximation of the mother electrocardiogram at the level *J*
$AS_{J}$	Approximation of a signal *S* at the level *j*
$DFECG_{J}$	Detail component of the fetal electrocardiogram at the level *J*
$DMECG_{J}$	Detail component of the mother electrocardiogram at the level *J*
$DS_{J}$	Detail component of a signal *S* at the level *J*
$d_{j,k}$	detail coefficient at the level *j* and the position *k*
$C_{j,k}$	Approximation coefficient at the level *j* and the position *k*
$ES_{j}$	The energy of a signal *S* at the level *j*
ES	The total energy of a signal *S*
Ent	The entropy of the signal
Ent(J)	The entropy of the signal at the level *J*
